# Exosomal miR-30d-5p of neutrophils induces M1 macrophage polarization and primes macrophage pyroptosis in sepsis-related acute lung injury

**DOI:** 10.1186/s13054-021-03775-3

**Published:** 2021-10-12

**Authors:** Yang Jiao, Ti Zhang, Chengmi Zhang, Haiying Ji, Xingyu Tong, Ran Xia, Wei Wang, Zhengliang Ma, Xueyin Shi

**Affiliations:** 1 Department of Anesthesiology, Nanjing Drum Tower Hospital, The Affiliated Hospital of Nanjing University Medical School, 321 Zhongshan Road, Nanjing, 210008 China; 2 Department of Anesthesiology and Intensive Care Unit, Xinhua Hospital, School of Medicine, Shanghai Jiaotong University, 1665 Kongjiang Road, Shanghai, 200092 China; 3 National Clinical Research Center of Kidney Diseases, Jinling Hospital, Nanjing University School of Medicine, Nanjing, China

**Keywords:** Sepsis-related acute lung injury, Neutrophil, Macrophage, Exosomes, miR-30d-5p, Pyroptosis

## Abstract

**Background:**

Polymorphonuclear neutrophils (PMNs) play an important role in sepsis-related acute lung injury (ALI). Accumulating evidence suggests PMN-derived exosomes as a new subcellular entity acting as a fundamental link between PMN-driven inflammation and tissue damage. However, the role of PMN-derived exosomes in sepsis-related ALI and the underlying mechanisms remains unclear.

**Methods:**

Tumor necrosis factor-α (TNF-α), a key regulator of innate immunity in sepsis-related ALI, was used to stimulate PMNs from healthy C57BL/6J mice in vitro. Exosomes isolated from the supernatant were injected to C57BL/6J wild-type mice intraperitoneally (i.p.) and then examined for lung inflammation, macrophage (Mϕ) polarization and pyroptosis. In vitro co-culture system was applied where the mouse Raw264.7 macrophages or bone marrow-derived macrophages (BMDMs) were co-cultured with PMN-derived exosomes to further confirm the results of in vivo animal study and explore the potential mechanisms involved.

**Results:**

Exosomes released by TNF-α-stimulated PMNs (TNF-Exo) promoted M1 macrophage activation after in vivo i.p. injection or in vitro co-culture*.* In addition, TNF-Exo primed macrophage for pyroptosis by upregulating NOD-like receptor 3 (NLRP3) inflammasome expression through nuclear factor κB (NF-κB) signaling pathway. Mechanistic studies demonstrated that miR-30d-5p mediated the function of TNF-Exo by targeting suppressor of cytokine signaling (SOCS-1) and sirtuin 1 (SIRT1) in macrophages. Furthermore, intravenous administration of miR-30d-5p inhibitors significantly decreased TNF-Exo or cecal ligation and puncture (CLP)-induced M1 macrophage activation and macrophage death in the lung, as well as the histological lesions.

**Conclusions:**

The present study demonstrated that exosomal miR-30d-5p from PMNs contributed to sepsis-related ALI by inducing M1 macrophage polarization and priming macrophage pyroptosis through activating NF-κB signaling. These findings suggest a novel mechanism of PMN-Mϕ interaction in sepsis-related ALI, which may provide new therapeutic strategies in sepsis patients.

**Supplementary Information:**

The online version contains supplementary material available at 10.1186/s13054-021-03775-3.

## Introduction

Sepsis is defined as a global health priority by the World Health Organization (WHO) and characterized by excessive inflammation in response to infection, with a reported death rate of 30–45% in hospitalized patients [1, 2]. Acute respiratory distress syndrome (ARDS) is the most common severe manifestation of multiple organ dysfunction syndrome and a significant factor contributing to the morbidity and mortality of sepsis [3].

Polymorphonuclear neutrophils (PMNs) are the most abundant leukocytes in mammals, which play a crucial role in the pathogenesis of sepsis-related acute lung injury (ALI) or ARDS [4]. Exosomes are small extracellular vesicles secreted by various cell types, with size ranging from 30 to 150 nm [5]. They can transfer a multitude of proteins and genetic material (including DNA, mRNA and microRNA [miRNA]) to target cells, playing a key role in cell-to-cell communications [6]. Animal studies have implicated the roles of PMN-derived exosomes in many chronic lung injuries, including chronic obstructive pulmonary disease (COPD), bronchopulmonary dysplasia and asthma [7, 8], but their role in sepsis-related ALI remains unclear.

In addition to PMNs, M1 macrophages, as a proinflammatory phenotype, also promoted the occurrence of sepsis-related ALI. M1 macrophages could be activated by circulating plasma exosomes, which further promote the secretion of proinflammatory cytokines, such as interleukin 1β (IL-1β), IL-12, IL-6 and tumor necrosis factor-α (TNF-α) [9, 10]. Recently, the pyroptosis of macrophage (Mϕ) has also been highlighted in sepsis-related ALI. Pyroptosis is a caspase-1-dependent proinflammatory cell death type [4, 11]. Caspase-1 activated by inflammasomes including NOD-like receptor 3 (NLRP3) inflammasomes initiates pyroptosis by cleaving gasdermin D (GSDMD, the core event in pyroptosis) to form pores in the plasma membrane, leading to cell swelling and membrane rupture, finally resulting in the leakage of mature forms of IL-1β and IL-18 out of cells [12, 13]. Pyroptotic macrophage-released danger signals or danger-associated molecular pattern molecules enhance inflammatory responses in sepsis-related ALI [14].

The crosstalk between PMNs and macrophages in regulating inflammation has been documented [15–18]. We previously reported that exosomes secreted from macrophages promoted neutrophil necroptosis following hemorrhagic shock [17]. Macrophage pyroptosis in sepsis could also be primed by neutrophil extracellular traps (NETs) [19]. However, the effects of PMN-derived exosomes on the behavior of macrophage and the underlying mechanisms in sepsis-related ALI are unknown.

Here, we firstly identified the role of PMN-derived exosomes in sepsis-related ALI by inducing macrophage M1 polarization and priming macrophage for pyroptosis. Bioinformatics analysis and further mechanistic studies revealed that PMN-derived exosomes transferred miR-30d-5p into macrophages and then activated NF-κB signaling pathway by inhibiting SOCS-1 (suppressor of cytokine signaling) and sirtuin 1 (SIRT1), which were both recognized as negative regulators of NF-κB signaling pathway previously [20, 21]. These findings suggest a previously unidentified pathway of PMN-Mϕ crosstalk, which could enhance macrophage activation and death, and subsequently exaggerate post-sepsis inflammation and induce lung injury.

## Materials and methods

### Animals

Wild type (WT) male C57BL/6J mice aged 6–8 weeks (Shanghai Sippr-BK Laboratory Animal Co., Ltd., Shanghai, China) were fed under a specific pathogen-free environment in Xinhua Hospital Animal Laboratory (Shanghai, China). All animal experiments were conducted under the rules approved by the Ethics Committee of Xinhua Hospital Affiliated to Shanghai Jiao Tong University School of Medicine (Approval No.: XHEC-F-2020-019).

### PMNs isolation and activation

PMNs were induced in the peritoneal cavity of the mice as previously described [22]. Briefly, mice were injected intraperitoneally (i.p.) with 1 ml 9% casein solution twice overnight and killed 3 h after the second injection to harvest the peritoneal lavage fluid (PLF), which was subsequently centrifuged, and the cell pellets were washed. PMNs were isolated by discontinuous density gradient centrifugation with two commercially available solutions (Histopaque-1077 and Histopaque-1119) of differential density (Sigma (St. Louis, MO; #11191 and #10771) according to the manufacturer’s instructions. The resulting cells consisted of 90% PMNs, and viability of the isolated PMNs was 95% as assessed by flow cytometry and Trypan blue staining, respectively.

After isolation, PMNs were suspended in complete culture medium (RPMI 1640 containing 10% exosome-free FBS, supplemented with 50 mg/ml penicillin/streptomycin) at a concentration of 10^6^ cells/ml. PMNs activation was induced upon 12-h incubation with 20 ng/mL TNF-α at 37 °C. An equal volume of phosphate buffered saline (PBS) to TNF-α was used as negative control.

### Exosome isolation and characterization

Exosomes were isolated from the supernatant of PMNs treated with PBS (PBS-Exo) or TNF-α (TNF-Exo) ex vivo using Total Exosome Isolation Reagent (#4484450; Thermo Fisher Scientific, Waltham, MA, USA). The detailed isolation procedure and the methods used to determine exosomal morphology, size distribution, and surface marker expression are described in Additional file 1.

### In vivo exosome administration to WT C57BL/6 mice

To explore exosome function in vivo, five WT C57BL/6 mice in each group were injected with PBS-Exo or TNF-Exo (300 μg/mouse) intraperitoneally using a 31-gauge insulin syringe, respectively. An equal volume of PBS was used as negative control. After 24 h, the obtained peritoneal lavage fluid was centrifuged, and peritoneal macrophages were detected by flow cytometry after gating with F4/80. To visualize changes in morphology and macrophage polarization, lung tissues were harvested and fixed in 4% paraformaldehyde for H&E and immunofluorescence staining. H&E staining was evaluated by a pathologist who was blinded to the experimental groups. To evaluate the lung injury, five independent random lung fields were evaluated per mouse for neutrophils in alveolar spaces, neutrophils in interstitial spaces, hyaline membranes, proteinaceous debris filling the airspaces, and alveolar septal thickening, and weighed according to the official American Thoracic Society workshop report on features and measurements of experimental ALI in animals [23]. The resulting injury score is a continuous value between 0 and 1. For immunofluorescence staining, paraffin-embedded lung tissues were sectioned, blocked with PBS containing 1% goat serum and 3% BSA, permeabilized with PBS/Triton 0.01%, and incubated with F4/80 and iNOS antibodies, and then with species-specific secondary antibodies coupled with Alexa Fluor Dyes. DNA was stained using DAPI. The sections were treated with autofluorescent quenching solution (#G1221; Servicebio, Wuhan, China) and mounted in Vectashield Mounting Media.

The in vivo miR-30d-5p inhibitors were transfected into the mouse lung through tail vein injection using the in vivo*-*jetPEI (Polyplus-transfection SA, New York, NY). Briefly, the miR-30d-5p inhibitor or negative control (50 μg, N/P ratio = 6, i.e., 0.12 μl of in vivo*-*jetPEI per μg nucleic acid) dissolved in 200 μl 5% glucose solution was injected into each mouse 1 day before exosome injection, according to the manufacturer’s protocol.

### In vitro co-culture experiments

Raw264.7 macrophages or BMDMs were treated with PMN-derived exosomes (100 μg/ml) at 37 °C for 24 h. To induce pyroptosis, macrophages were primed with exosomes for 24 h before stimulation with 5 mM ATP (#HY-B2176; MedChemExpress, Monmouth Junction, NJ, USA) or 20 μM nigericin (#HY-100381; MedChemExpress) for 2 h.

For miR-30d-5p inhibition, Raw264.7 macrophages were transfected with microRNA control or miR-30d-5p inhibitor (Guangzhou Ribobio Corporation, Guangzhou, China) at a concentration of 50 nM using Lipofectamine 3000 for 24 h prior to be co-cultured with exosomes as per the manufacturer’s instructions. For miR-30d-5p overexpression, Raw264.7 macrophages were transfected with microRNA control or miR-30d-5p mimic at a concentration of 50 nM using Lipofectamine 3000 for 48 h.

### Flow cytometry analysis of M1 polarization and pyroptosis

Macrophages were centrifuged and resuspended in PBS for FACS analysis. According to the manufacturer’s instructions, anti-CD11c (#117307; BioLegend, San Diego, CA, USA), anti-CD86 (#105106; BioLegend), anti-CD206 (#141706; BioLegend) and anti-F4/80 (#123116; BioLegend) antibodies were used for fluorescent staining. Isotype antibody controls were used to exclude nonspecific staining. Data were obtained using a CytoFLEX flow cytometer (Beckman, USA). Programmed cell death was analyzed with apoptosis detection kit (#559763; BD Biosciences, Franklin Lakes, NJ). Macrophages were incubated with Annexin-V and PI for 15 min at room temperature in the dark and then analyzed by flow cytometry. Cells double-stained positive for Annexin V and PI were considered as undergoing programmed death. Cell pyroptosis was detected by two-color flow cytometry. Macrophages were incubated with Alexa Fluor 488-labeled caspase-1 FLICA (#ICT098; ImmunoChemistry, Bloomington, MN, USA) at 37 °C for 1 h. After being fixed with 4% paraformaldehyde, cells were stained with TMR red-labeled In-Situ Cell Death Detection reagent (#12156792910; Roche Applied Science, Indianapolis, IN, USA), following the manufacturer’s instructions. Double-stained cells were identified as pyroptotic cells. Background and autofluorescence were determined by a control antibody with the same isotype staining.

### Sequencing of exosomal miRNA and data analysis

Total RNA was extracted from PBS-Exo/TNF-Exo using the miRNeasy Serum/Plasma Kit (Qiagen, Valencia, CA, USA). The final ligation PCR products were sequenced using the BGISEQ-500 platform (BGI Group, Shenzhen, China). After acquiring the raw data, the differentially expressed miRNAs were calculated using the *t* test. Those with ≥ twofold upregulation and a *P* value < 0.05 were regarded as significantly different. A heat map was generated using the R 3.5.3 software. Pathway analysis was conducted using the Kyoto Encyclopedia of Genes and Genomes (KEGG) pathway database. The 20 most enriched pathways related to signaling transduction are listed and were used to reveal the most associated pathways.

### Quantitative real-time PCR (RT-qPCR)

Total RNA isolation was performed using TRIzol following the manufacturer’s instruction (TAKARA, Japan). mRNA was reverse transcribed using PrimeScript RT reagent Kit (#RR036; TAKARA, Tokyo, Japan), and PCR was conducted using TB Green™ Premix Ex Taq™ (Tli RNaseH Plus) (#RR420A; TAKARA) and QuantStudio™ 3 System (Applied Biosystems). Data were normalized to the expression of GAPDH. Primer sequences are shown in Additional file 1: Table S1.

For exosomal miRNA quantification, total RNA was extracted from 200 μl PMN-derived exosomes using miRNeasy Serum/Plasma kit (#217184; Qiagen, Valencia, CA, USA). RNA pellets were resuspended in 14 μl RNase-free water. Twelve microliters of RNA solution were used for reverse transcription, according to the protocol of miScript RT Kit (#218161; Qiagen). miRNA expression was quantified using a miScript SYBR Green PCR Kit (#218075; Qiagen). qPCR analysis was also performed for miR-30d-5p expression in cells or the lung tissue. Briefly, RNA was extracted by TRIzol reagent (TAKARA, Japan) and RT-qPCR was conducted using the Mir-X miRNA qRT-PCR SYBR Kit (#638316; TAKARA). Relative expression was calculated using the comparative cycle threshold (Ct) method (2^−ΔΔCT^) normalized to U6. The miRNA qPCR primers were purchased from Guangzhou Ribobio Corporation.

### Luciferase assay

The 3′-UTR of the SOCS-1/SIRT1 sequence containing the predicted miR-30d-5p binding sites and its mutant was cloned into the plasmid vector and transfected into HEK293 cells. A renilla luciferase vector was co-transfected in all transfections described to monitor transfection efficiency. All luciferase results were reported as relative light units: the average of the photinus pyralis firefly activity observed was divided by the average of the activity recorded from the renilla luciferase vector.

### Mouse model of cecal ligation and puncture (CLP)

The CLP mouse model was prepared as previously described [22]. Mice were anesthetized with ketamine (50 mg/kg) and xylazine (5 mg/kg) via i.p. injection. After disinfection, a 1 cm midline laparotomy was made in the abdomen. The cecum was then exteriorized, and ligated below the cecal valve, and punctured with an 18-gauge needle to induce sepsis. A small drop of cecal content was extruded. The cecum was then returned to the peritoneal cavity and the abdominal incision closed with sutures. Mice were resuscitated with (5 ml/100 g) saline. Sham animals underwent the same surgical procedures without cecum ligation and puncture. The in vivo miR-30d-5p inhibitors were transfected into the mouse lung through tail vein injection using the in vivo*-*jetPEI one day before CLP surgery, according to the manufacturer’s protocol. At 24 h after surgery, the animals were euthanized with phenobarbital overdose (100 mg/kg body weight), followed by collection of the lung tissues as described previously.

### Statistics

The normal distribution of the data was tested using the Shapiro–Wilk test. Normally distributed data are presented as means ± SEM. Comparisons between 2 groups were performed by the 2-tailed Student’s t test. Multiple group comparisons were performed by one-way ANOVA followed by Tukey's multiple comparisons test with GraphPad Prism 8 software. Comparison of survival rates between groups was performed using Log-rank test. A value of *P* < 0.05 was considered statistically significant.

## Results

### TNF-Exo induces lung injury by affecting M1 macrophage activation and pyroptosis in vivo

TNF-α is a potent inducer of inflammatory response and a key regulator of innate immunity in sepsis-related ALI, which is often used to activate neutrophils [24–26]*.* Thus, PMNs from healthy C57BL/6J mice were treated with PBS (PBS-Exo) or stimulated with TNF-α (TNF-Exo) ex vivo to isolate exosomes. Morphologically, transmission electron microscopy (TEM) showed that these isolated microvesicles displayed a round, cup-shaped morphology (Additional file 2: Fig. S1A), with a diameter about 70 nm (Additional file 2: Fig. S1B). Western blot further confirmed high expression of exosome-specific markers CD9, CD63 and TSG101 (Additional file 2: Fig. S1C).

**Fig. 1:**
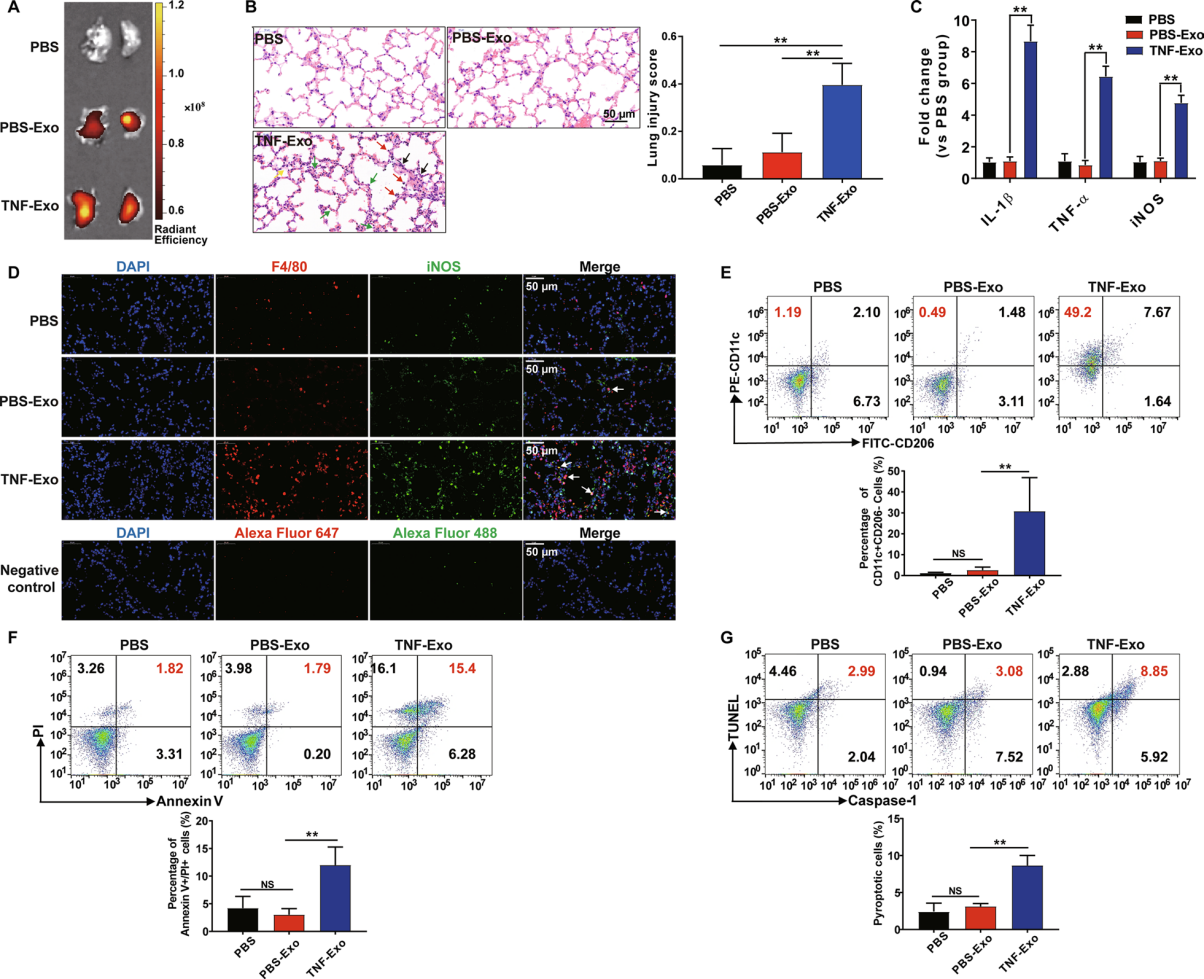
TNF-Exo induces lung injury by affecting M1 macrophage activation and pyroptosis in vivo. WT C57BL/6 mice were administered with PBS-Exo/TNF-Exo (300 μg/mouse) intraperitoneally for 24 h. An equal volume of PBS was used as negative control. **a** Ex vivo fluorescence signals in the lungs of mice injected i.p. with Dil-labeled exosomes. **b** Evaluation of lung histology by H&E staining (magnification × 400). Green arrows indicate neutrophils in the alveolar and interstitial space, red arrows indicate alveolar macrophages, yellow arrows indicate hyaline membranes, and black arrows indicate thickening of the alveolar walls. Scale bar, 50 μm. **c** Detection of inflammatory cytokine mRNA (IL-1β, TNF-α) and iNOS mRNA expression in the lung tissues by RT-qPCR. **d** Representative images of direct immunofluorescence staining of DNA (blue), F4/80 (red) and iNOS (green) in the lung sections, and white arrows indicate iNOS positive macrophages. Negative control represents immunofluorescence images stained with species-specific secondary antibodies coupled with Alexa Fluor Dyes alone. Scale bar, 50 μm. **e** Flow cytometry detection of CD11c and CD206 expression on peritoneal macrophage (PMϕ) after being gated with macrophage marker F4/80. **f** Representative flow cytometry plots of Annexin V/PI staining of PMϕ, and analysis of Annexin V/PI double-stained cells by dying. **g** Representative flow cytometry plots and quantitation of PMϕ pyroptosis (Caspase-1/TUNEL double-positive cells). One-way analysis of variance with Tukey's multiple comparisons test was used for the analysis. Graphs represent means ± SEM, *n* ≥ 3; *P < 0.05, **P < 0.01 compared within two groups

TNF-Exo was injected i.p. to C57BL/6J WT mice to determine whether TNF-Exo could induce lung inflammation. Fluorescence imaging showed that TNF-Exo accumulated in the lung tissue after TNF-Exo injection (Fig. 1a). Histological lesions were observed in the mouse lung after the administration of TNF-Exo compared with PBS-Exo (Fig. 1b). Meanwhile, the pro-inflammatory mediators (iNOS, IL-1β and TNF-α) in the lung tissue were highly expressed and the number of local M1 macrophages (iNOS + F4/80 + cells) was significantly increased (Fig. 1c, d), suggesting that the lung inflammation induced by TNF-Exo was in parallel with the macrophage inflammatory activity. Furthermore, we found that TNF-Exo significantly increased the proportion of M1 macrophage (CD11c + CD206-) and dying cells (Annexin V + PI +) in peritoneal macrophage (PMϕ) as assessed by flow cytometry (Fig. 1e, f). To further determine the type of PMϕ death, PMϕ was detected for nuclear fragmentation, caspase-1 activation (the characteristics of pyroptosis) by flow cytometry and the result showed ~ 8% pyroptotic PMϕ at 24 h after TNF-Exo injection (Fig. 1g). All these data indicate that TNF-Exo in vivo could induce pulmonary inflammation potentially due to M1 macrophage activation and pyroptosis.

### TNF-Exo promotes M1 macrophage activation and primes macrophage pyroptosis through NF-κB pathway in an in vitro co-culture model

**Fig. 2:**
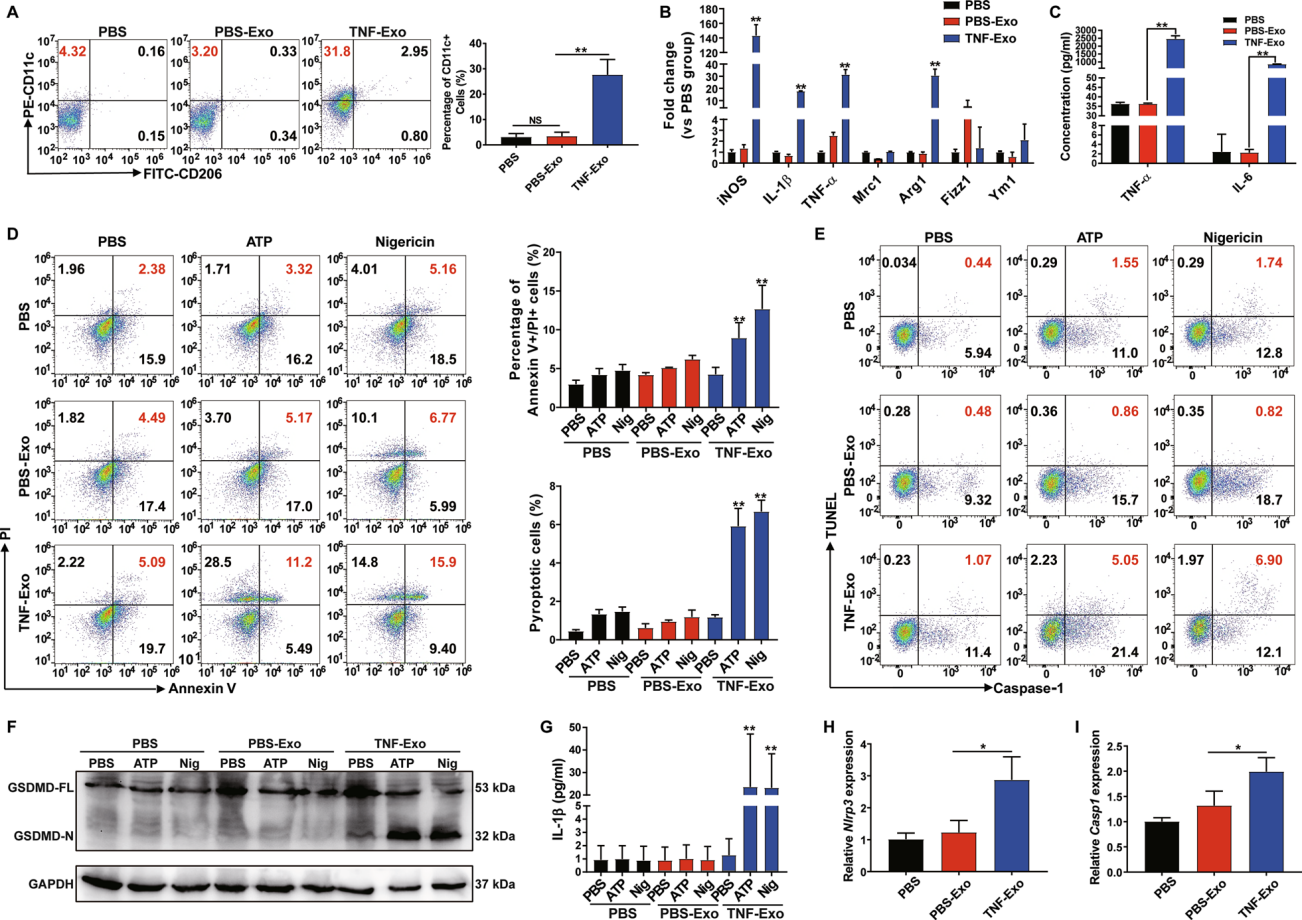
TNF-Exo promotes M1 macrophage activation and primes macrophage pyroptosis through NF-κB pathway in an in vitro co-culture model.** a**–**c** Treatment of Raw264.7 macrophages with PBS-Exo/TNF-Exo for 24 h. An equal volume of PBS was used as negative control. **a** Flow cytometry detection of CD11c and CD206 expression on Raw264.7 macrophages. **b** Detection of expression levels of iNOS, IL-1β, TNF-α, Mrc1, Arg1, Fizz1 and Ym1 mRNA by RT-qPCR. **c** Detection of the concentration of inflammatory cytokines (IL-6, TNF-α) in the supernatant of Raw264.7 macrophages by ELISA. **d–g** Stimulation of Raw264.7 macrophages with PBS/PBS-Exo/TNF-Exo for 24 h and treatment with PBS, 5 mM ATP or 20 mM nigericin for 2 h. **d** Flow cytometry evaluation of macrophage death by Annexin-V and PI double-staining. **e** Flow cytometry evaluation of macrophage pyroptosis by Caspase-1 and TUNEL double-staining. **f** Cleavage of GSDMD by western blot. GSDMD-FL: full-length GSDMD, GSDMD-N: N-terminal cleavage products of GSDMD. **g** Analysis of culture supernatants for IL-1β secretion by ELISA. **h–i** Treatment of Raw264.7 macrophages with PBS/PBS-Exo/TNF-Exo for 24 h, and detection of NLRP3 and caspase-1 mRNA expression by RT-qPCR. One-way analysis of variance with Tukey's multiple comparisons test was used for the analysis. Graphs represent means ± SEM, *n* ≥ 3; *P < 0.05, **P < 0.01 compared within two groups

Using an in vitro co-culture system, macrophages were co-cultured with PMN-derived exosomes to further confirm the results of the in vivo animal study. First, we observed the direct transfer of exosomes between PMNs and Mϕ, as evidenced by Mϕ internalization of PMN-derived exosomes using confocal microscopy (Additional file 2: Fig. S2A). In addition, no matter in primary cell or cell line of macrophage, TNF-Exo could promote macrophage M1 polarization (Fig. 2a–c, Additional file 2: S2B–S2D). However, TNF-Exo did not promote macrophage death or pyroptosis directly; after adding ATP/nigericin, pyroptotic cell death of TNF-Exo-primed macrophages was significantly upregulated (Fig. 2d–e). ATP/nigericin is often used as positive controls for NLRP3 inflammasome activation by initiating the assembly of inflammasomes [27]. Activated NLRP3 inflammasomes splice IL-1β precursor for maturation and secretion, and cleave GSDMD to initiate pyroptosis. The cleaved GSDMD N-terminus and IL-1β secretion were increased in TNF-Exo plus ATP or nigericin group as assessed by Western blot and ELISA, respectively (Fig. 2f–g). Besides, TNF-Exo increased NLRP3 and caspase-1 mRNA expressions in macrophages as measured by RT-qPCR (Fig. 2h–i). These data suggest that TNF-Exo only served as a priming signal to upregulate NLRP3 inflammasome expression, while the second signals, such as ATP or nigericin, were required to finally induce macrophage pyroptosis in vitro.

Next, we wanted to determine through which pathway TNF-Exo promoted M1 macrophage activation and primed macrophage pyroptosis in vitro by co-culturing macrophages with exosomes by proteomics (Additional file 3). The proteomic results were consistent with the above finding that TNF-Exo significantly upregulated the expression of M1 macrophage marker (inducible nitric oxide synthase, iNOS) and the expression of NLRP3 complexes (NLRP3 and Caspase-1) (Additional file 2: S2E). Enrichment pathway analysis showed that NF-κB signaling pathway was within the 20 most enriched pathways (Additional file 2: S2F), and Western blot demonstrated that NF-κB activity was increased in TNF-Exo-treated macrophages (Additional file 2: S2G). The proinflammatory transcription factor NF-κB is known to regulate M1 macrophage polarization and NLRP3 inflammasome expression [20, 28]. Taken together, the above results suggested that TNF-Exo upregulated NF-κB signaling activity to promote M1 macrophage activation and prime macrophage for pyroptosis.

### miRNA analysis of PMN-derived exosomes

**Fig. 3:**
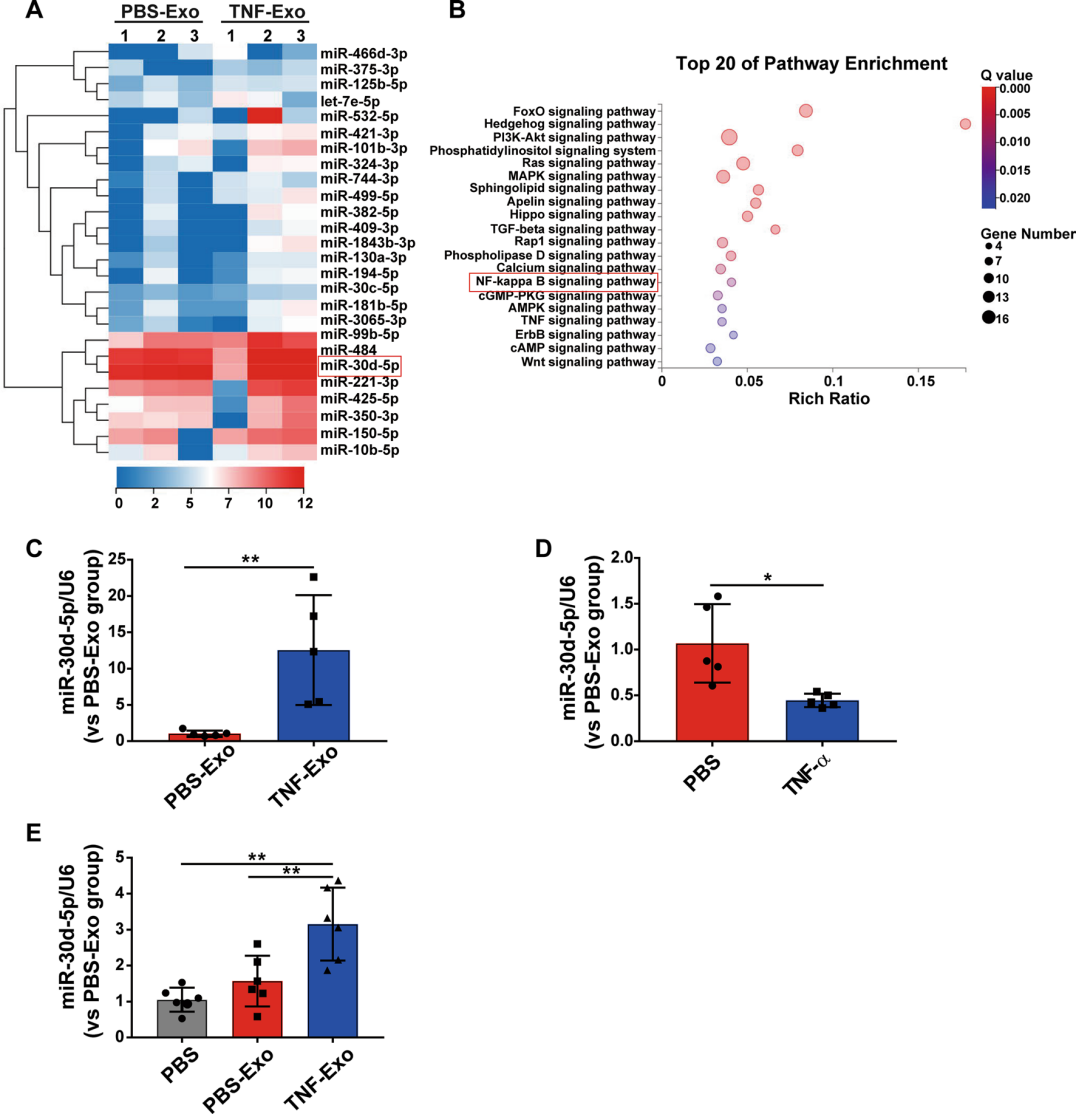
miRNA analysis of PMN-derived exosomes. **a** Heat map of exosomal miRNA-seq (*n* = 3). The fluorescence intensity of 26 differentially expressed miRNAs (≥ twofold) is illustrated from high (red) to low (blue). **b** Kyoto Encyclopedia of Genes and Genomes (KEGG) analysis on differentially expressed exosomal miRNAs; the 20 most enriched pathways related to signaling transduction are shown. The rich ratio indicates the number of genes in the miRNA target list over the total genes in the respective canonical pathway. **c–e** Expression of miR-30d-5p in PMN-derived exosomes, in PMNs stimulated with PBS/TNF-α and in recipient Raw264.7 macrophages treated with PBS/PBS-Exo/TNF-Exo by RT-qPCR. Student’s t test (**c, d**) or one-way analysis of variance with Tukey's multiple comparisons test (E) was used for the analysis. Graphs represent means ± SEM, *n* ≥ 3; *P < 0.05, **P < 0.01 compared within two groups

Knowing that exosomes from septic shock patients can convey miRNAs related to pathogenic pathways, which may represent a novel mechanism for intercellular communication during sepsis [29], we subsequently screened PMN-derived exosomes for miRNAs and detected 26 miRNAs. It was found that they increased by ≥ twofold in TNF-Exo as compared those in PBS-Exo (Fig. 3a, Additional file 2: Table S1). Enrichment pathway analysis was also performed to identify the most enriched pathways related to signaling transduction for these 26 miRNAs, and the data showed that NF-κB signaling pathway was within the 20 most enriched pathways (Fig. 3b).

Next, we searched the literature and found that miR-30d-5p expression was positively correlated with NF-κB signaling pathway [30]. We thus used RT-qPCR and found that the expression of miR-30d-5p in TNF-Exo was significantly higher than that in PBS-Exo (Fig. 3c). It was surprisingly found that the level of miR-30d-5p in PMNs was decreased after TNF-α stimulation, indicating that miR-30d-5p relocated from the cellular compartment to exosomes (Fig. 3d). Interestingly, macrophages exhibited a higher level of miR-30d-5p after being cultured with TNF-Exo in vitro (Fig. 3e). These results indicate that TNF-α could enhance miR-30d-5p loading into exosomes from PMNs and transferring to recipient macrophages. Thus, we hypothesized that TNF-Exo may transfer miR-30d-5p into macrophages and then activate NF-κB signaling pathway.

### TNF-Exo activates NF-κB signaling pathway in macrophage via miR-30d-5p

**Fig. 4:**
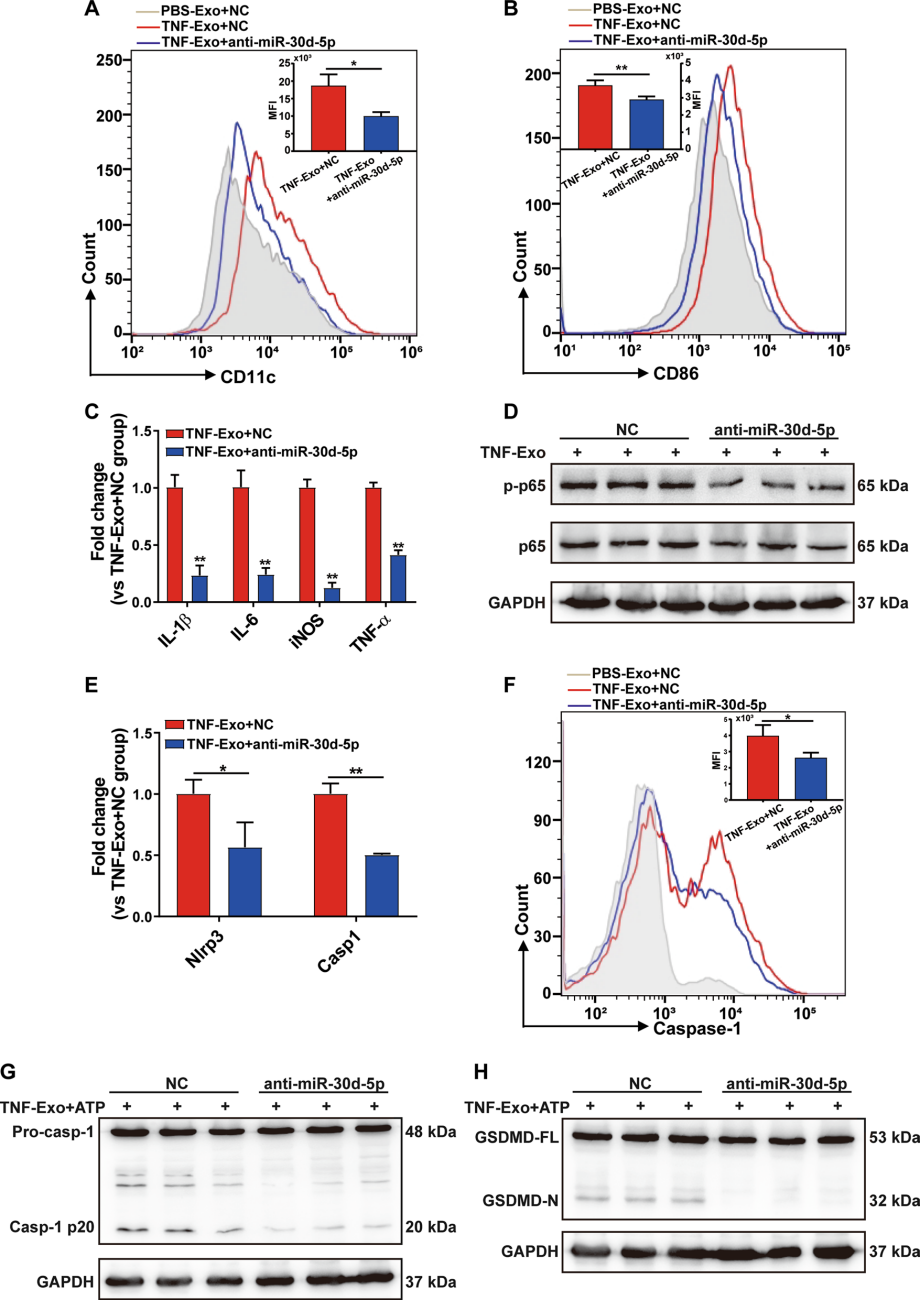
TNF-Exo activates NF-κB signaling pathway in macrophage via miR-30d-5p.** a–e** Prior to co-culturing with TNF-Exo for 24 h, Raw264.7 macrophages were transfected with negative control (NC) or miR-30d-5p inhibitors (anti-miR-30d-5p) for 24 h. **a, b** Flow cytometry detection of CD11c and CD86 expression. **c** Detection of inflammatory cytokine mRNA (IL-6, IL-1β, TNF-α) and iNOS mRNA expression by RT-qPCR. **d** Western blot of NF-κB p-p65 and p65 in Raw264.7 macrophages. **e** Detection of NLRP3 and caspase-1 mRNA expression by RT-qPCR. **f–h** Transfection of Raw264.7 macrophages with negative control (NC) or miR-30d-5p inhibitors for 24 h, followed by stimulation with TNF-Exo plus ATP. **f** Flow cytometry detection of Alexa Fluor 488-labeled caspase-1 FLICA expression. **g** Western blots of pro-caspase-1 (Pro-casp-1) and activated/cleaved caspase-1 (Casp-1 p20) in whole-cell lysates of Raw264.7. **h** Cleavage of GSDMD by immunoblotting. Student’s t test was used for analysis. Graphs represent means ± SEM, *n* ≥ 3; *P < 0.05, **P < 0.01 compared within two groups

To test the above hypothesis, we transfected Raw264.7 macrophages with miR-30d-5p inhibitors prior to culturing with TNF-Exo. It was found that transfection of miR-30d-5p inhibitors reversed the upregulation of M1 macrophage markers and pro-inflammatory cytokines induced by TNF-Exo (Fig. 4a–c). In addition, inhibition of miR-30d-5p significantly decreased NF-κB p-p65 protein expression in recipient macrophages treated with TNF-Exo (Fig. 4d).

Moreover, treatment of Raw264.7 macrophages with a miR-30d-5p inhibitor prior to culturing with TNF-Exo decreased the mRNA level of NLRP3 and caspase-1 (Fig. 4e). Intracellular caspase-1 activation was also measured by flow cytometry and Western blot. Transfection of miR-30d-5p inhibitors exhibited a significant suppressive effect on caspase-1 activation in response to TNF-Exo plus ATP (Fig. 4f–g). The cleaved GSDMD N-terminus upregulated by TNF-Exo plus ATP stimulation was also inhibited by miR-30d-5p inhibitors (Fig. 4h). These data show that exosomal miR-30d-5p promoted M1 macrophage activation and primed macrophage for pyroptosis through activating NF-κB signaling pathway.

### Exosomal miR-30d-5p activates NF-κB in macrophage via targeting SOCS-1 and SIRT1

**Fig. 5:**
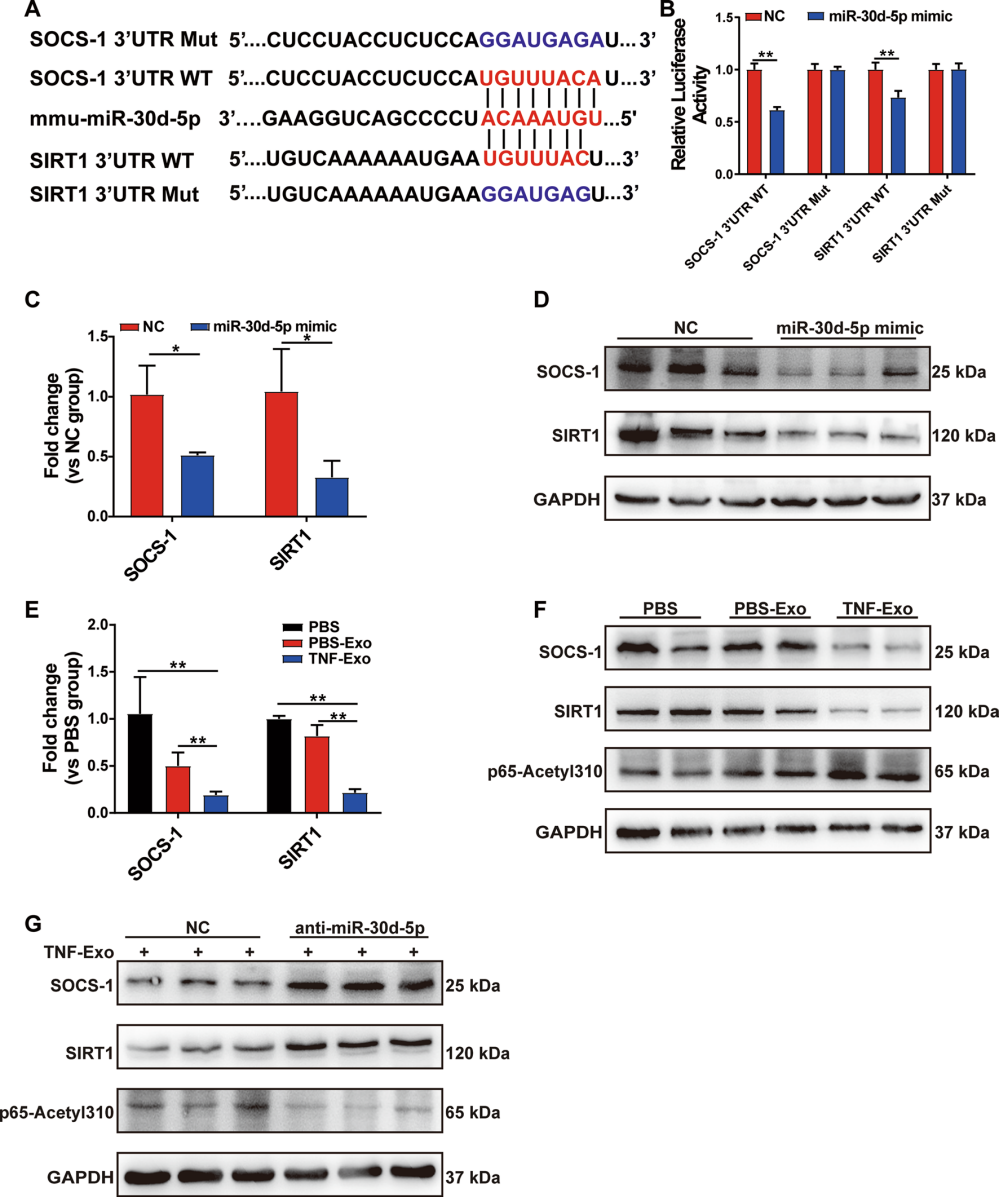
Exosomal miR-30d-5p activates NF-κB in macrophage via targeting SOCS-1 and SIRT1. **a** Sequence alignment between miR-30d-5p and its putative binding sites (in red letters) in the SOCS-1/SIRT1 3′-UTR. Mutation of the miR-30d-5p target sites (in blue letters) is also shown. **b** Detection of the relative luciferase activities of WT and Mut SOCS-1/SIRT1 reporters by luciferase reporter assay, using Renilla luciferase vector as the internal control. RT-qPCR analysis of relative SOCS-1/SIRT1 mRNA level (**c**) and Western blot (**d**) of SOCS-1 and SIRT1 in Raw264.7 macrophages transfected with miR-30d-5p mimics as indicated. **e, f** Treatment of Raw264.7 macrophages with PBS/PBS-Exo/TNF-Exo for 24 h. **e** Detection of mRNA levels of SOCS-1 and SIRT1 by RT-qPCR. **f** Western blot analysis of SOCS-1, SIRT1 and p65-Acetyl 310. **g** Prior to co-culturing with TNF-Exo for 24 h, Raw264.7 macrophages were transfected with control or miR-30d-5p inhibitors for 24 h. The expression levels of SOCS-1, SIRT1 and p65-Acetyl 310 in Raw264.7 cells were measured by Western blot. Student’s t test (**b, c**) or one-way analysis of variance with Tukey's multiple comparisons test (**e**) was used for the analysis. Graphs represent means ± SEM, *n* ≥ 3; *P < 0.05, **P < 0.01 compared within two groups

Next, we sought to understand the mechanism through which miR-30d-5p activated NF-κB signaling pathway. Bioinformatics analysis showed that SOCS-1 (suppressor of cytokine signaling) and sirtuin 1 (SIRT1) were putative target genes for miR-30d-5p, and also negative regulators of NF-κB signaling pathway. As predicted by Targetscan in our study, miR-30d-5p may conserve the binding sites in the 3’ UTR of SOCS-1 and SIRT1 (Fig. 5a). To validate this bioinformatic prediction, we conducted a dual luciferase reporter assay and found that luciferase activity was markedly reduced by miR-30d-5p overexpression in SOCS-1/SIRT1 3’-UTR WT group, but not in 3′-UTR Mut group (Fig. 5b). In Raw264.7 macrophages, overexpression of miR-30d-5p suppressed both the mRNA and protein levels of SOCS-1 and SIRT1 (Fig. 5c–d). All these results demonstrate that SOCS-1 and SIRT1 were the direct target genes of miR-30d-5p.

To determine whether exosomal miR-30d-5p targeted SOCS-1/SIRT1 in macrophages, we examined the expression levels of SOCS-1 and SIRT1 and found that both mRNA and protein expressions of SOCS-1 and SIRT1 were decreased in TNF-Exo-treated macrophages (Fig. 5e–f), while miR-30d-5p inhibitors reversed the expression of SOCS-1 and SIRT1 (Fig. 5g). In addition, previous studies indicated that SIRT1 reduced NF-κB activity by decreasing the acetylation level of lysine 310 of the NF-κB p65 subunit [21]. Indeed, the enhancement of p65 lysine 310 acetylation after TNF-Exo was observed in our study (Fig. 5f), which decreased after miR-30d-5p inhibition (Fig. 5g). These results suggest that exosomal miR-30d-5p targeted SOCS-1 and SIRT1 in macrophages and subsequently activated NF-κB partly by increasing acetylation of lysine 310 of p65.

### miR-30d-5p inhibition alleviates TNF-Exo or CLP-induced lung injury

**Fig. 6:**
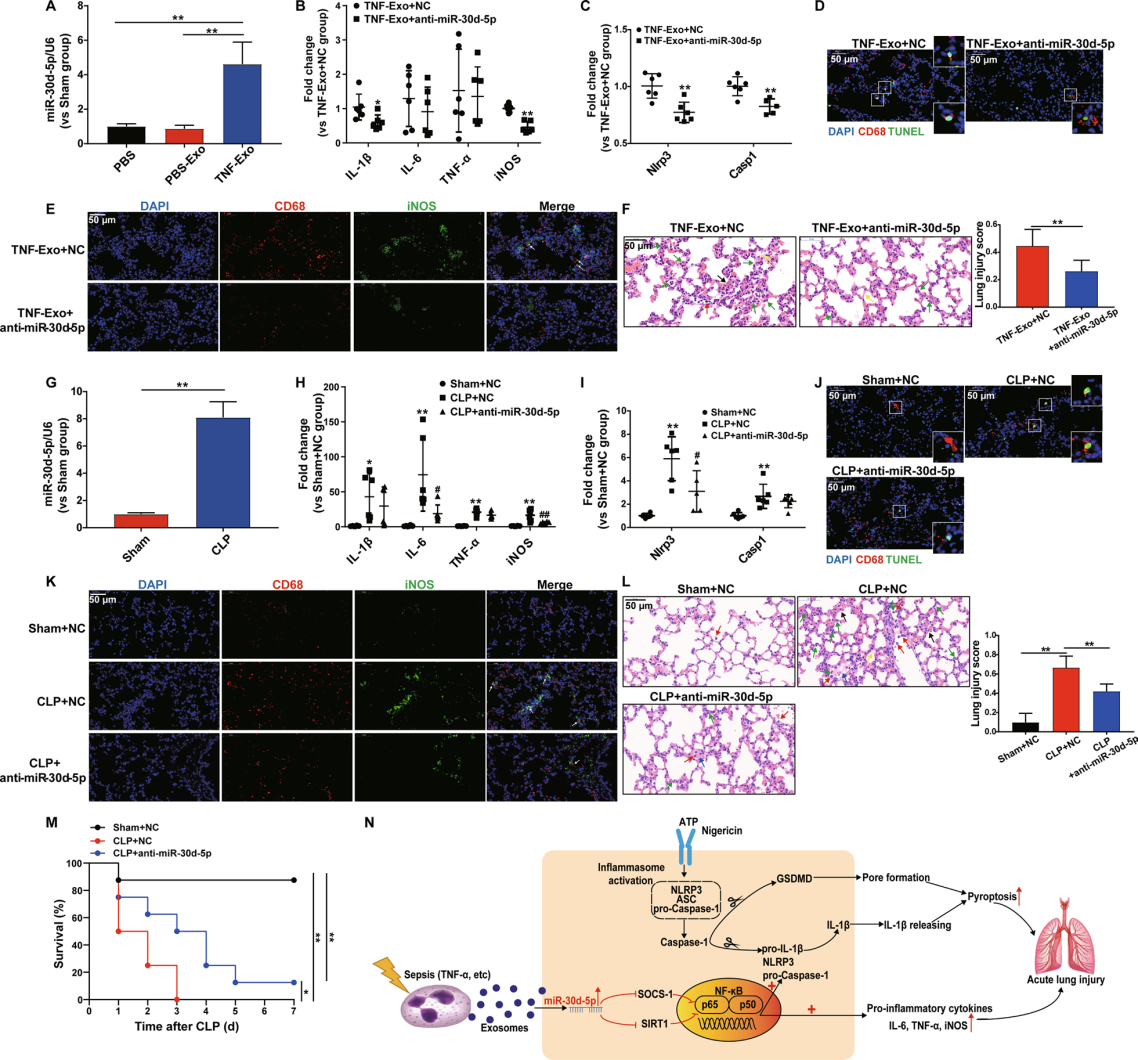
miR-30d-5p inhibition alleviates TNF-Exo or CLP-induced lung injury. Mice were injected with TNF-Exo (300 μg/mouse) intraperitoneally (**a–f**) or subjected to sham or CLP (**g–l**) for 24 h. The miR-30d-5p inhibitor or negative control (NC) was transferred into each mouse 1 day before TNF-Exo injection or CLP surgery. Relative expression levels of miR-30d-5p (**a, g**), inflammatory cytokine mRNA (IL-6, IL-1β, TNF-α) and iNOS mRNA (**b, h**), NLRP3 and caspase-1 mRNA expression (**c, i**) in the lung tissues were measured by RT-qPCR. **d, j** Representative images of direct immunofluorescence staining of DNA (blue), CD68 (red) and TUNEL (green) in the lung sections. Scale bar, 50 μm. **e, k** Representative images of direct immunofluorescence staining of DNA (blue), CD68 (red) and iNOS (green) in the lung sections, and white arrows indicate iNOS positive macrophages. Scale bar, 50 μm. **f, l** Evaluation of lung histology by H&E staining (magnification × 400). Green arrows indicate neutrophils in the alveolar and interstitial space, red arrows indicate alveolar macrophages, yellow arrows indicate hyaline membranes, blue arrows indicate proteinaceous debris filling the airspaces, and black arrows indicate thickening of the alveolar walls. Lung injury scores were assessed. Scale bar, 50 μm. Student’s t test or one-way analysis of variance with Tukey's multiple comparisons test was used for the analysis. **m** Survival rate of CLP mice with or without miR-30d-5p inhibition (*n* = 8) and log-rank test was used for the analysis. **n** Schematic representation of the mechanism by which neutrophil-derived exosomes induce M1 macrophage polarization and prime macrophage pyroptosis in sepsis-related ALI. Graphs represent means ± SEM, *n* ≥ 3; *P < 0.05, **P < 0.01 compared within two groups (**h, i** ^*,**^P compared with Sham + NC group, ^#,##^*P* compared with CLP + NC group)

We next investigated the functional role of miR-30d-5p in TNF-Exo in vivo. It was found that miR-30d-5p expression was significantly increased in the lung tissue after TNF-Exo i.p. injection (Fig. 6a). Next, miR-30d-5p inhibitors or scrambled negative control were administered via the tail vein of mice before TNF-Exo injection, and the expression levels of proinflammatory cytokines and NLRP3 inflammasomes in the lung tissues were tested. The results showed that miR-30d-5p inhibitors significantly decreased the levels of IL-1β, iNOS, NLRP3, caspase-1 mRNA expressions following TNF-Exo administration (Fig. 6b–c). In addition, inhibition of miR-30d-5p decreased TNF-Exo-induced M1 macrophage activation and macrophage death in the lung (Fig. 6d, e), as well as the histological lesions (Fig. 6f).

In addition, the CLP mouse model was applied to mimic sepsis to further confirm the roles of miR-30d-5p in vivo. Likewise, miR-30d-5p expression was also significantly increased in the lung tissues of CLP mice (Fig. 6g). The decline in IL-6, iNOS, NLRP3 mRNA expression (Fig. 6h, i), the decrease in M1 macrophage activation and macrophage death in the lung (Fig. 6j, k), and the alleviation of lung injury were also observed in the CLP model with miR-30d-5p inhibition (Fig. 6l). Notably, the survival rate in CLP mice with miR-30d-5p inhibition was significantly higher than that without inhibition (Fig. 6m). All these data demonstrated that inhibition of miR-30d-5p could improve both TNF-Exo and CLP-induced lung injury through suppressing M1 macrophage activation and macrophage death.

## Discussion

It was found in our study that exosomal miR-30d-5p from PMNs induced macrophage M1 polarization and primed macrophages pyroptosis by activating NF-kB signaling via targeting SOCS-1 and SIRT1. In addition, we discovered a previously unidentified role of PMN-Mϕ interaction in promoting inflammation in sepsis-related ALI.

In the early stage of sepsis, neutrophils are thought to be the primary innate immune cells that causing damage to host tissues [31]. In addition to releasing important cytokines, chemokines, ROS and NETs, some studies suggested that PMN-derived exosomes were a new subcellular entity, working as a fundamental link between PMN-driven inflammation and tissue damage [8]. TNF-α plays a central role in the pathogenesis of sepsis and is an early regulator of the immune response [32]. Previous studies [33, 34] showed that TNF-α and IL-1β produced by macrophages activated neutrophils during sepsis, and high concentrations of TNF-α and IL-1β have been reported in BALF from ARDS patients. Therefore, in this study, we used TNF-α to activate PMNs from healthy mice and isolated exosomes from the supernatant.

Macrophages have been shown to be the recipient cells for exogenous exosomes and in direct contact with peripheral serum exosomes [35]. A recent study showed that peripheral serum exosomes promoted M1 macrophage polarization and inflammation during sepsis-related ALI, but the study did not address the cellular origin of the circulating exosomes [36]. Our results showed that exosomes isolated from the supernatant of PMNs stimulated with TNF-α promoted M1 macrophage activation both in vivo and in vitro. We also observed that TNF-Exo resulted in a significant lung inflammatory response, suggesting that exosomes released from TNF-α-activated PMNs are a kind of pro-inflammatory exosomes and play an important role in sepsis-related ALI.

In addition, we observed that ATP/nigericin significantly upregulated pyroptotic cell death in TNF-Exo-primed macrophages. Induction of pyroptotic cell death in vitro usually needs two signals: the priming signal and the secondary signal. The priming signal upregulates NLRP3 inflammasome and pro-IL-1β expression levels through the transcription factor NF-κB. After the priming phase, the secondary signal, such as ATP/nigericin, initiates the assembly of several protein complexes, including NLRP3, apoptosis-associated speck-like protein containing CARD (ASC) and pro-caspase-1, by regulating the formation of the ASC pyroptosome and splicing of caspase-1 into its active form [27]. Based on our results, TNF-Exo served as a priming signal to increase NLRP3 inflammasome expression through activating NF-κB signaling pathway, which still required the secondary signal to finally induce pyroptotic cell death. High concentrations of extracellular ATP have been implicated in multiple in vivo inflammatory responses, including lung inflammation and fibrosis, systemic inflammation and tissue damage during endotoxemia [37, 38]. Almost all mammalian cells, including myeloid cells, platelets, leukocytes, epithelial and endothelial cells, can release ATP in response to stimulation [39], which may explain why TNF-Exo could promote macrophage pyroptosis in vivo, as TNF-Exo or cytokines upregulated by TNF-Exo may stimulate other cells to release high concentrations of ATP. However, the exact mechanisms need to be further addressed.

Exosomally transferred miRNAs have emerged as novel regulators of cellular function. miRNA sequencing and literature search in our study suggest that miR-30d-5p may be the functional molecule within TNF-Exo. Our study here reported a novel function of miR-30d-5p in exosomes as a regulator of macrophage polarization and pyroptosis. The exosome-mediated inflammatory pathway may be a new mechanism responsible for the development of sepsis-related ALI by promoting PMN-Mϕ communication.

Finally, we further demonstrated the role of miR-30d-5p in TNF-Exo and CLP-induced lung injury. The expression level of miR-30d-5p was significantly increased in the lung after TNF-Exo administration, suggesting that miR-30d-5p could be transferred to the lung tissue via exosomes. miR-30d-5p loss-of-function markedly reduced M1 macrophage activation and death in the lung, and ameliorated lung injury, indicating that miR-30d-5p contributed to TNF-Exo-induced lung injury. Furthermore, inhibition of miR-30d-5p was found to be highly related to the improvement of lung injury and survival rate in the experimental sepsis model, which may provide a novel molecular target for the treatment of sepsis-related ALI.

There were several limitations in our study. Firstly, more studies about the correlations of miR-30d-5p with clinical parameters such as oxygenation index and mortality of sepsis-related ARDS patients are required to make our conclusions more informative and reliable. Secondly, we found that TNF-Exo in vivo could promote macrophage pyroptosis and TNF-Exo in vitro needed a second signal to finally induce macrophage pyroptosis, based on which raised the hypothesis that TNF-Exo or cytokines increased by TNF-Exo may stimulate other cells to release high concentrations of ATP in vivo, which served as a second signal to induce TNF-Exo-primed macrophage pyroptosis. However, the exact mechanisms need to be further addressed. Lastly, although specially inhibition of miR-30d-5p in PMNs in vivo is not easily manipulated, administration of imR-30d-5p inhibitors via the tail vein of mice before CLP in our study did exhibit a protective effect on lung injury and survival, suggesting that miR-30d-5p may represent a new therapeutic target for the progression of sepsis-related ALI.

## Conclusions

The data obtained in our study demonstrated that exosomal miR-30d-5p derived from PMNs could promote lung inflammation by enhancing M1 macrophage polarization and priming macrophage pyroptosis. Modulating the cross-talk between PMNs and macrophages attenuated tissue inflammation and injury during sepsis-related ALI, highlighting its potential as a therapeutic strategy in sepsis-related ARDS.

## Supplementary Information

**Additional file 1**. Supplementary methods

**Additional file 2**. Supplementary results

**Additional file 3**. Proteomic data

## Data Availability

The datasets generated and analyzed during the current study are available from the corresponding author on reasonable request.

## Abbreviations

PMNs: Polymorphonuclear neutrophils
ALI: Acute lung injury
TNF-α: Tumor necrosis factor-α
Mϕ: Macrophage
BMDMs: Bone marrow-derived macrophages
NLRP3: NOD-like receptor 3
NF-kB: Nuclear factor kB
SOCS-1: Suppressor of cytokine signaling
SIRT1: Sirtuin 1
CLP: Cecal ligation and puncture
ARDS: Acute respiratory distress syndrome
miRNAs: MicroRNAs
IL-1β: Interleukin 1β
GSDMD: Gasdermin D
NETs: Neutrophil extracellular traps
WT: Wild type
PLF: Peritoneal lavage fluid
PMϕ: Peritoneal macrophage
RPMI: Roswell park memorial institute
FBS: Fetal bovine serum
PBS: Phosphate buffer saline
Exos: Exosomes
H&E: Hematoxylin and eosin
iNOS: Inducible nitric oxide synthase
RT-PCR: Real-time polymerase chain reaction
TEM: Transmission electron microscopy
ASC: Apoptosis-associated speck-like protein containing CARD
