# Supplementary material for: Exosomal miR-30d-5p of neutrophils induces M1 macrophage polarization and primes macrophage pyroptosis in sepsis-related acute lung injury
Source: Crit Care. 2021 Oct 12;25:356. doi: 10.1186/s13054-021-03775-3 (PMC8507252; doi:10.1186/s13054-021-03775-3)
Supplement: Supplementary file 1 — Additional file 1. Supplementary methods [file 13054_2021_3775_MOESM1_ESM.docx]

**Supplementary Methods**

**Exosome isolation and characterization**

Exosomes were isolated from the supernatant of PMNs stimulated ex vivo using Total Exosome Isolation Reagent. Briefly, the culture medium was centrifuged at 2000 g for 10 min to remove cells and debris, centrifuged the supernatant at 10,000 g for 30 min to further remove debris, and then filtered by a 0.22 μm filter (Millipore). The Total Exosome Isolation Reagent for cell culture medium was added following the manufacturer’s instructions. The final pellet containing exosomes were resuspended in PBS.

The morphology of the exosomes was characterized using transmission electron microscopy. The size distribution of exosomes was assessed by NanoSight tracking analysis. The surface markers of exosomes (CD9, CD63 and TSG101) were identified by western blot, loading with the same amount of exosome protein (60 μg).

**Transmission electron microscopy**

After isolation of exosomes, the pellets were fixed with 2% glutaraldehyde in 0.1 M phosphate buffer (pH 7.4). The fixed pellets were placed on 100-mesh, carbon-coated, formvar-coated nickel grids treated with poly-L-lysine for 30 min. After washing the samples with several drops of PBS, samples were incubated with drops of buffered 1% glutaraldehyde for 5 min and then washed several times with drops of distilled water. Afterward, samples were negatively stained with drops of Millipore-filtered aqueous 4% uranyl acetate for 5 min. Stain was blotted dry from the grids with filter paper, and samples were allowed to dry. The microscopy images were captured by a JEOL JEM-1400 transmission electron microscope operating at 120 kV.

**Proteomics**

After 24-h coculture with PBS-Exo/TNF-Exo, BMDMs were stored at -80 °C prior to use. After protein extraction, trypsin digestion and tandem mass tag (TMT) labeling, the sample was then fractionated by high pH reverse-phase HPLC using an Agilent 300 Extend C18 column (5 μm particles, 4.6 mm ID, 250 mm in length). The resulting peptides were analyzed by a Q Exactive Plus Hybrid Quadrupole-Orbitrap mass spectrometer (Thermo Scientific), and then subjected to nanospray-ionization followed by tandem mass spectrometry (MS/MS) in a Q Exactive Plus (Thermo Scientific) coupled online to the UPLC. The resulting MS/MS data were processed using the Mascot search engine (v.2.3.0). Tandem mass spectra were searched against the mouse protein sequence database from UniProt. Trypsin/P was specified as the cleavage enzyme, allowing up to 2 missing cleavages. The mass error was set to 10 ppm for precursor ions, and 0.02 Da for fragment ions. Carbamidomethylation of cysteine residues was specified as a fixed modification, and methionine oxidation was specified as a variable modification. For the protein quantification method, TMT-6-plex was selected in Mascot. The false discovery rate (FDR) was adjusted to < 1%, and the peptide ion score was set at ≥ 20.

**Western blot analysis**

Cells were lysed in ice-cold RIPA Lysis buffer supplemented with protease inhibitor cocktail, and the protein concentration was determined by BCA assay. Cell lysates were then boiled in SDS sample buffer and resolved on 10-12% SDS-PAGE gel. Immunoblots were incubated overnight with primary antibodies against CD9 (#A19027; ABclonal, Wuhan, China), CD63 (#A5271; ABclonal), TSG101 (#A1692; ABclonal), NLRP3 (#A5652; ABclonal), IL-1β (#31202; Cell Signaling Technology, Danvers, MA, USA), Acetyl-NF-κB p65 (Lys310) (#12629S; Cell Signaling Technology), P-p65 (#3033S; Cell Signaling Technology), p65 (#8242S; Cell Signaling Technology), caspase-1 (#A0964; ABclonal), GSDMD (#ab209845; Abcam, Cambridge, MA, USA), SOCS-1 (#A7754; ABclonal), SIRT1 (#9475T; Cell Signaling Technology) and GAPDH (#5174; Cell Signaling Technology). Immunoblots were examined using an ECL detection reagent (#WBULS0500; Millipore Corporation, Billerica, MA, USA).

**Fluorescence imaging of Dil-exosomes**

Exosomes were isolated from the supernatant of PMNs stimulated *ex vivo* and every 10 μg of isolated exosomes were incubated with 1 μl Dil labeling solution (#V22885, Thermo Fisher Scientific) for 30 min at 37℃. Then, the Dil-exosomes were precipitated using Total Exosome Isolation Reagent according to the manufacturer’s protocol. Dil-exosomes were then resuspended in 200 µL PBS buffer and injected into mice (*i.p.*). After 24 h, the lungs were dissected for *ex vivo* evaluation and the biodistribution of Dil-exosomes in the lungs was monitored using IVIS Spectrum In Vivo Imaging System (PerkinElmer, Waltham, MA, USA). Filters allowing excitation at 560 nm and collection of emission at 590 nm were used to obtain ideal images.

**References**

1. Xu F, Zhang C, Zou Z, Fan E, et al: Aging-related Atg5 defect impairs neutrophil extracellular traps formation. Immunology 2017; 151(4):417-432

**Table 1** **Primers for quantitative RT-PCR.**

| Primer | Forward (5’-3’) | Reverse (5’-3’) |
| --- | --- | --- |
| IL-1β | TGCCACCTTTTGACAGTGATG | AAGGTCCACGGGAAAGACAC |
| IL-6 | AAAGAGTTGTGCAATGGCAATTCT | AAGTGCATCATCGTTGTTCATACA |
| iNOS | CAGATCGAGCCCTGGAAGAC | CTGGTCCATGCAGACAACCT |
| TNF-α | CATCTTCTCAAAATTCGAGTGACAA | TGGGAGTAGACAAGGTACAACCC |
| Arg1 | ACATTGGCTTGCGAGACGTA | ATCACCTTGCCAATCCCCAG |
| Mrc1 | CTCTGTTCAGCTATTGGACGC | TGGCACTCCCAAACATAATTTGA |
| Fizz1 | ACTTTGATGGCCTCAACCTG | AATGATTCCTGCTCCTGTGG |
| Ym1 | CCCTTCTCATCTGCATCTCC | AGTAGCAGTCATCCCAGCA |
| Nlrp3 | ATTACCCGCCCGAGAAAGG | TCGCAGCAAAGATCCACACAG |
| Casp1 | ACAAGGCACGGGACCTATG | TCCCAGTCAGTCCTGGAAATG |
| SOCS-1 | CACTCACTTCCGCACCTTCC | CAGCCGGTCAGATCTGGAAG |
| SIRT1 | TCGGCTACCGAGGTCCATA | CCGCAAGGCGAGCATAGATA |
| GAPDH | GCATGGCCTTCCGTGTTC | GATGTCATCATACTTGGCAGGTTT |
