# Supplementary material for: Exosomal miR-30d-5p of neutrophils induces M1 macrophage polarization and primes macrophage pyroptosis in sepsis-related acute lung injury
Source: Crit Care. 2021 Oct 12;25:356. doi: 10.1186/s13054-021-03775-3 (PMC8507252; doi:10.1186/s13054-021-03775-3)
Supplement: Supplementary file 2 — Additional file 2. Supplementary results [file 13054_2021_3775_MOESM2_ESM.docx]

**Supplementary results**

**
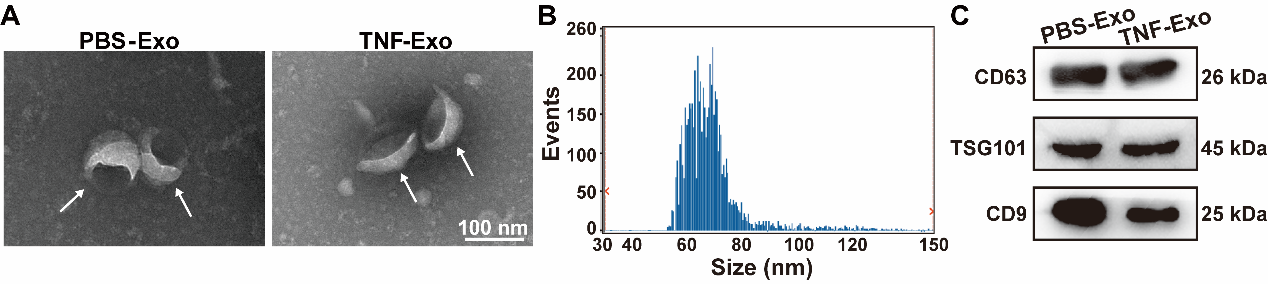
Figures**

**Supplementary Figure S1.** Characterization of exosomes isolated from the supernatant of PMNs stimulated ex vivo. **A** Electron micrograph of exosomes (indicated by arrows) isolated from the supernatant of PMNs treated with PBS (PBS-Exo) or 20 ng/mL TNF-α (TNF-Exo) for 12 h. Scale bar, 100 nm. **B** Measurement of exosome size distribution by NanoSight tracking analysis. **C** The expressions of CD63, TSG101 and CD9 in exosomes by western blot loaded with equal amounts of exosome protein (60 μg).


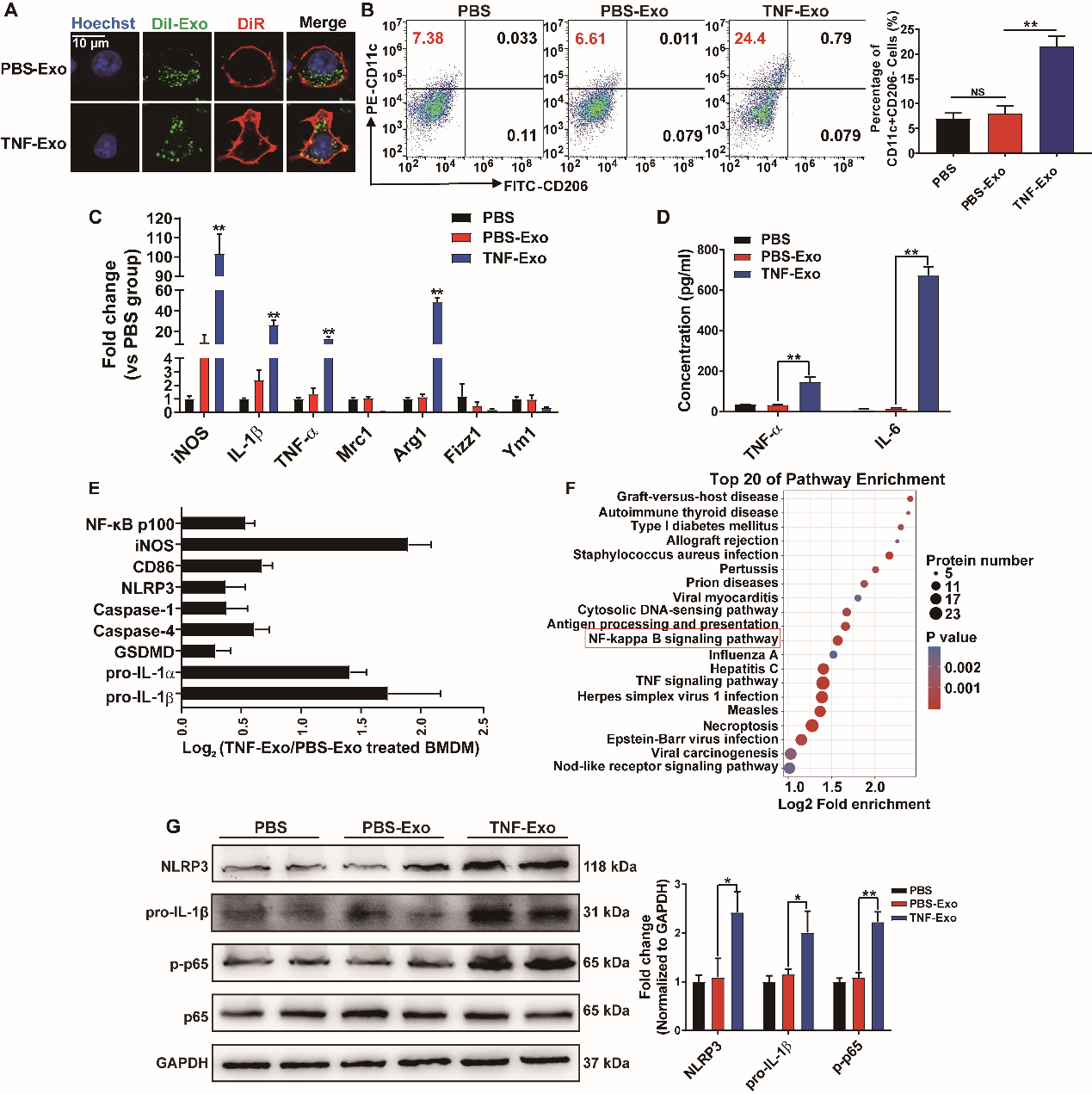
**Supplementary Figure S2.** Bone marrow-derived macrophages (BMDMs) were treated with PBS-Exo/TNF-Exo for 24 h. **A** Immunofluorescence images showing BMDMs incubated with Dil-labeled exosomes (green) for 3 h. Cell membranes and nuclei were counterstained with DiR (red) and Hoechst (blue) respectively. Scale bar, 10 μm. **B** Flow cytometry detection of CD11c and CD206 expression on BMDMs. **C** Detection of expression levels of iNOS, IL-1β, TNF-α, Mrc1, Arg1, Fizz1 and Ym1 mRNA by real-time PCR. **D** Detection of the concentration of inflammatory cytokines (IL-6, TNF-α) in the supernatant of BMDMs by ELISA. **E** BMDMs for tandem mass tags (TMT)-based proteomic quantification. **F** Enrichment pathway analysis showed that NF-κB signaling pathway was within the 20 most enriched pathways. **G** Western blot of NLRP3, pro-IL-1β, NF-κB p-p65 and p65 in BMDMs after co-culturing with exosomes. Student’s t test or one-way analysis of variance with Tukey's multiple comparisons test was used for the analysis. Graphs represent means ± SEM, n ≥ 3; **P* < 0.05, ***P* < 0.01 compared within two groups.

**Table 1 Differentially expressed miRNAs (≥ 2-fold) by miRNA sequencing (n = 3).**

| **miRNA** | **Fold change**  **(TNF-Exo/PBS-Exo)** | | ***P* value** | | **Regulated Type** |
| --- | --- | --- | --- | --- | --- |
| mmu-miR-466d-3p | | 2.258 | | ＜0.001 | Up |
| mmu-miR-375-3p | | 2.182 | | ＜0.001 | Up |
| mmu-miR-125b-5p | | 2.116 | | ＜0.001 | Up |
| mmu-let-7e-5p | | 2.000 | | ＜0.001 | Up |
| mmu-miR-532-5p | | 357.3 | | ＜0.001 | Up |
| mmu-miR-421-3p | | 2.241 | | ＜0.001 | Up |
| mmu-miR-101b-3p | | 2.298 | | ＜0.001 | Up |
| mmu-miR-324-3p | | 2.375 | | ＜0.001 | Up |
| mmu-miR-744-3p | | 3.384 | | ＜0.001 | Up |
| mmu-miR-499-5p | | 6.269 | | ＜0.001 | Up |
| mmu-miR-382-5p | | 3.756 | | ＜0.001 | Up |
| mmu-miR-409-3p | | 3.320 | | ＜0.001 | Up |
| mmu-miR-1843b-3p | | 11.20 | | ＜0.001 | Up |
| mmu-miR-130a-3p | | 3.261 | | ＜0.001 | Up |
| mmu-miR-194-5p | | 2.085 | | ＜0.001 | Up |
| mmu-miR-30c-5p | | 2.500 | | ＜0.001 | Up |
| mmu-miR-181b-5p | | 2.830 | | ＜0.001 | Up |
| mmu-miR-3065-3p | | 3.885 | | ＜0.001 | Up |
| mmu-miR-99b-5p | | 3.268 | | ＜0.001 | Up |
| mmu-miR-484 | | 2.289 | | ＜0.001 | Up |
| mmu-miR-30d-5p | | 2.072 | | ＜0.001 | Up |
| mmu-miR-221-3p | | 2.160 | | ＜0.001 | Up |
| mmu-miR-425-5p | | 2.047 | | ＜0.001 | Up |
| mmu-miR-350-3p | | 2.183 | | ＜0.001 | Up |
| mmu-miR-150-5p | | 2.638 | | ＜0.001 | Up |
| mmu-miR-10b-5p | | 2.220 | | ＜0.001 | Up |
